# Mechanical and Thermal Properties of Phosphoric Acid Activated Geopolymer Materials Reinforced with Mullite Fibers

**DOI:** 10.3390/ma15124185

**Published:** 2022-06-13

**Authors:** Qingxin Wei, Yang Liu, Huirong Le

**Affiliations:** The Future Lab, Tsinghua University, Beijing 100084, China; weiqingxin@mail.tsinghua.edu.cn (Q.W.); liu-yang18@mails.tsinghua.edu.cn (Y.L.)

**Keywords:** geopolymer, composites, fibers, mechanical properties, thermal resistance

## Abstract

This article investigates several important properties, such as thermal resistance, mechanical properties, and phase evolution, of geopolymer ceramics reinforced with mullite fibers. This particular fiber reinforcing geopolymer composites was prepared from kaolinite and mullite fibers with phosphoric acid as activator. X-ray diffraction (XRD), thermogravimetry and differential scanning calorimetry, Fourier-transform infrared spectroscopy, and scanning electron microscopy were used to determine the phase evolution and strengthening mechanisms. With the addition of mullite fibers, the mechanical properties increased by at least 20%. The optimum flexural strength exceeded 13 MPa. It was found that mullite fibers had desirable interface bonding with this type of geopolymer, promoting both crack deflection and fiber pullout strengthening mechanisms. This was correlated with a significant strengthening effect of the fibers. The linear shrinkage after heat treatment at 1150 °C~1550 °C was investigated and correlated with XRD analyses. The addition of mullite fibers reduced the linear shrinkage significantly up to 1350 °C. The large linear shrinkage above 1450 °C was correlated with the decomposition and melting of the AlPO_4_ phase.

## 1. Introduction

It was first reported in 1978 by French scientist Joseph Davidovits that naturally occurring aluminosilicates, such as kaolinite, could be transformed at ambient temperature within an astonishingly short time into three-dimensional aluminosilicate mineral polymer [1,2]. This has attracted a lot of attention since the discovery. Scientists have widely studied the reaction mechanisms and physicochemical properties of the materials, and found more and more applications in building materials, refractories, and so on [3,4]. The material is also used to fix industrial and nuclear wastes containing heavy metals or radiative elements, which makes it particularly useful in the field of environmental protection [5]. However, its insufficient mechanical properties and high-temperature stability have hindered its wider applications [6].

Alkaline activated geopolymers have been widely studied, but suffer from insufficient thermal resistance due to the formation of a glassy phase containing alkaline elements. Compared with alkali activated geopolymers, phosphoric acid activated geopolymers have superior high-temperature properties without the interference of alkaline elements [7]. However, there have been few studies on phosphoric acid activated geopolymers so far. Unlike the rapid curing of alkali activated geopolymers, the curing speed of phosphoric acid activated geopolymers is relatively slow and usually needs to be carried out above room temperature, adding more cost to manufacturing [8].

As geopolymer materials can generally complete the initial curing within one day without the need for calcination, this consumes less energy than other refractories and the manufacturing process is simpler. Potential applications of thermal resistant geopolymer ceramics include furnace refractory boards, casting mold cores, fire-retardant concrete, runway pavement, etc. These applications require good thermal resistance and sufficient mechanical properties. However, the existing geopolymer materials suffer from insufficient mechanical strength and thermal resistance. Fiber reinforcement is regarded as an efficient method to improve both mechanical and thermal resistance of ceramics. A considerable number of fibers are selected as the reinforcing phase to improve the properties of geopolymers, including natural fibers such as cotton fiber [9], wood fiber [10], hemp fiber [11], and so on [12,13]. There have also been studies using ceramic fibers as the reinforcing phase, such as silicon carbide fibers [14,15]. Fang et al. [16] investigated the property evolution of geopolymer composites with BN-coated SiC whiskers at elevated temperatures. The composites exhibited improved high-temperature strength up to 900 °C. Yan et al. [17] investigated the high-temperature mechanical properties of C_f_-SiC_f_ reinforced alkaline based geopolymer composites, which also showed improved high-temperature resistance up to 800 °C. However, the mechanical properties deteriorated above 1000 °C.

This paper is focused on phosphoric acid activated geopolymer, because of its superior high-temperature performance [18,19]. Short fibers are employed to overcome its higher probability of brittle failure due to insufficient mechanical strength and temperature-induced degradation. Mullite short fibers were used as the reinforcing phase because of their good temperature resistance [20], low cost, and similar compositions to the geopolymer matrix material studied.

## 2. Materials and Methods

### 2.1. Raw Materials

Kaolinite was purchased from Sigma-Aldrich Inc, with a specific surface area of 10.162 m^2^/g according to the supplier, and the chemical compositions of kaolinite and metakaolinite determined by XRF are shown in Table 1. Instead of distilled water, tap water was used along with industrial phosphoric acid (85%) with a view to industrial applications. Mullite fibers were short fibers sourced from Rayjohn Refractories Ltd. (Huzhou, China). Mullite fiber has an average diameter of 3–5 μm, average length of 2 mm, alumina content of about 75%, and a melting point of about 1850 °C, according to the supplier.

### 2.2. Sample Preparation

#### 2.2.1. Fiber Dispersion

It is a widely used method to use short fibers as a reinforcing method for a wide range of materials [21], but fiber dispersion is a common problem to be solved. If the fibers are agglomerated and knotted, this easily causes defects in the composite and reduces the mechanical properties. The as-received mullite fibers were in the form of agglomerates. The initial idea to mix the fibers directly with metakaolinite did not break the agglomerates of fibers. After many explorations, we developed an effective process to disperse the fibers, as described below.

The first step of fiber dispersion was to mix a certain amount of fiber with water and stir it quickly in one direction. In this study, typically the fiber weight was 5~20 g and water 300 mL. In this step, a magnetic stirrer in the laboratory was used. After about 30 min, a uniformly dispersed fiber suspension was obtained, and then a certain amount of kaolinite was gradually added to the fiber suspension under continuous stirring. A uniform slurry of the fiber, kaolinite, and water was obtained. Finally, the slurry was filtered by vacuum-assisted filtering to avoid agglomeration of fibers. The obtained cake was dried and broken by ball milling. The milling time was not be too long, typically ball milling at 150 rpm for 10 min and then at 300 rpm for 10 min to ensure the mullite fibers were not broken. Then, the powder was calcined at 800~900 °C [22,23] for 1 h to obtain a mixture containing metakaolinite and mullite fibers.

It has been previously reported that when the molar ratio of Al_2_O_3_:H_3_PO_4_ = 1:1.3, phosphate-based geopolymer yields the best strength [18]. The recommended weight ratio of metakaolinite to liquid phase is about 1:1 for ease of processing. As such, phosphoric acid was diluted to a specific concentration and added to the powder to obtain both a desirable Al_2_O_3_:H_3_PO_4_ ratio and metakaolinite to liquid ratio. Since the fiber is generally considered to be the part that does not participate in the reaction, phosphoric acid was added in proportion to the amount of metakaolinite only. The compositions of the composites with various fiber content are summarized in Table 2.

#### 2.2.2. Geopolymer Composite Preparation

**Figure 1:**
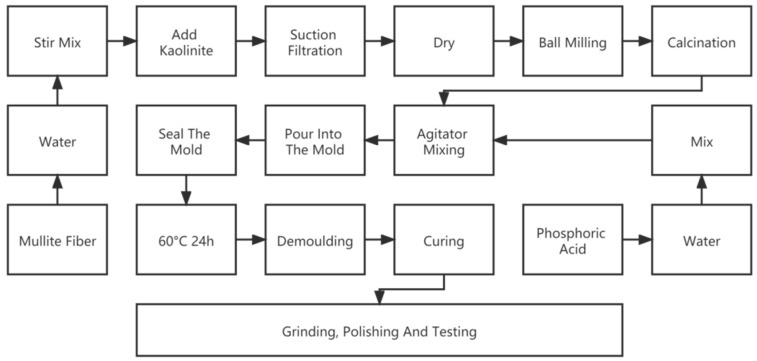
Preparation process of mullite fiber geopolymer composite.

The obtained solid and liquid phases were mixed in a mixer (MSK-SFM-7; Kejing; Shenyang, China) for 30 min, then poured into a mold made by 3D printing, sealed, and placed on the shaking table at 120 rpm for 1 h to remove bubbles. The materials were cured for 24 h in an autoclave at 60 °C, then removed from the mold and kept in humidity above 80% at room temperature for 7/28 days. Two types of samples were made for three-point bending and compression tests to obtain flexural strength and compressive strength, respectively. The process flowchart is shown in Figure 1.

### 2.3. Microstructural Analysis and Mechanical Testing

**Figure 2:**
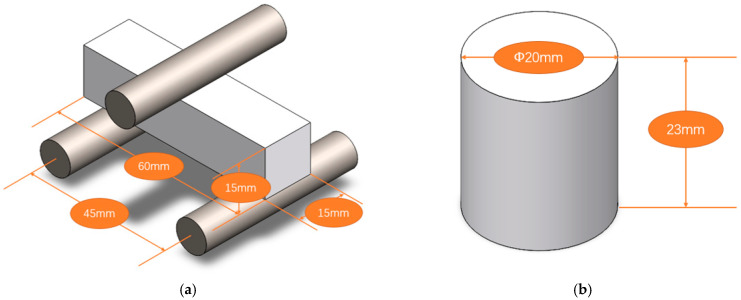
Specimen size for mechanical property testing: (**a**) configuration of three-point bending, (**b**) compressive strength test sample dimensions.

The three-point bending and compression test samples are shown in Figure 2. Cylindrical specimens were used for compressive strength testing, while rectangular specimens were used for three-point bending testing, with a crosshead speed of 1 mm/min using a mechanical testing machine (50ST; Tinius Olsen; Horsham, PA, USA). As shown in Figure 2, the size of the rectangular sample for three-point bending was 60 mm × 15 mm × 15 mm, and the sample for the compression test was a cylinder with a diameter of 20 mm and a height of 23 mm. The peak load of six parallel test pieces was used to calculate the flexural strength by formula σ_f_ = 3FL/2bh^2^, where σ_f_ is the flexural strength (MPa), *F* is the critical load (N), *L* is the span (mm), *b* is the width of the specimen (mm), and *h* is the height of the specimen (mm). The compressive strength was determined by σ_c_ = 4F/πd^2^, where σ_c_ is the compressive strength (MPa), *F* is the critical load (N), and *d* is the diameter of the specimen (mm). Each group of compression tests included 3 samples.

Scanning electron microscopy (SEM; Merlin, Zeiss Company, Oberkochen, Germany) was adopted to observe the microstructures of the mullite fiber reinforced geopolymer composites fabricated under various conditions and the fractured cross-sections resulting from the three-point bending test.

To determine the crystalline phases of the specimens, X-ray diffraction (XRD) tests were carried out (D8 Advance, Bruker Optics, Ettlingen, Germany) using scanning range of 10° to 80°. X-ray fluorescence spectrometry (XRF; ARL PERFORM X; Thermo Fisher; Shanghai, China) was used to determine the chemical composition of the powder sample.

Thermogravimetry (TG; STA8000, PerkinElmer, Walthamm, MA, USA) coupled with FTIR (Spectrum 3, PerkinElmer, Walthamm, MA, USA) was used to simultaneously obtain the thermal dynamic information and the functional groups of the specimens. Nitrogen with a flow rate of 20 mL/min was injected during the heating process, and the temperature was increased to 1600 °C at a heating rate of 10 °C/min.

High-temperature treatment was carried out in a muffle furnace (KSL-1750X; Kejing; Shenyang, China), heated at 5 °C/min and held at the target temperature for 1 h, then cooled to room temperature to obtain the treated sample. Rectangular samples with dimensions of about 30 mm× 15 mm× 15 mm were used for high-temperature shrinkage testing and microstructural analysis. Cutting and polishing of samples were carried out with a metallographic sample cutting machine (SYJ-50; Kejing; Shenyang, China) and a grinding/polishing machine (UNIPLO-802; Kejing; Shenyang, China).

## 3. Results and Discussions

### 3.1. Interfaces between Geopolymer and Fiber

**Figure 3:**
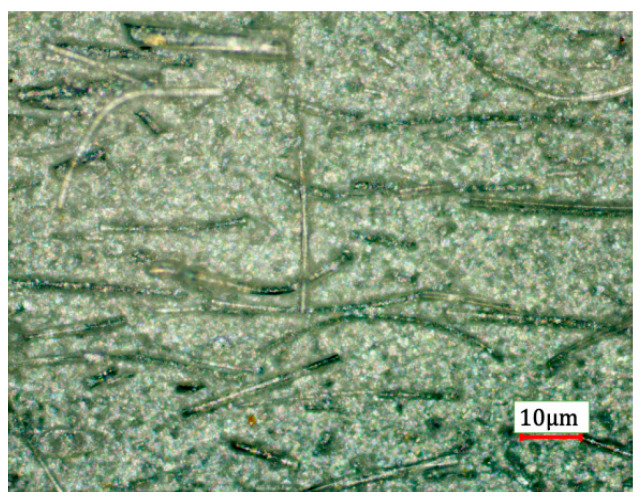
The fibers were evenly dispersed in the slurry.

Mullite is one of the phases of geopolymer produced after calcination above 1150 °C [24], but the proportion of mullite produced by polymerization is small. On the one hand, the addition of mullite fiber increases the weight proportion of the highest melting point component in the composite. Furthermore, the fibrous structure can hinder the shrinkage of the matrix at high temperature. The dispersion of the fibers is critical to the properties of composites. A typical SEM image of the dispersion of fibers in the green body is shown in Figure 3, indicating excellent dispersion of mullite fibers using the process described in Section 2.1.

**Figure 4:**
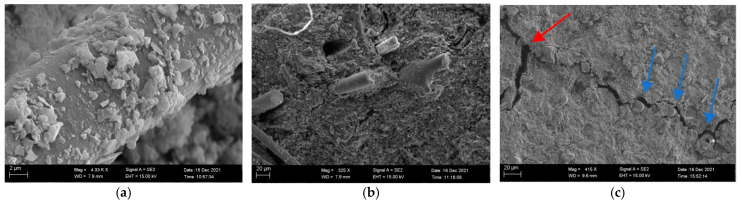
SEM photos of the interface between mullite fiber and geopolymer matrix: (**a**) geopolymer grains on mullite fibers, (**b**) fiber pullout, (**c**) crack deflection.

Fractal surfaces after three-point bending tests were also examined using SEM. It was found that geopolymer particles had grown on the surface of the mullite fibers (c.f. Figure 4a), which confirmed that mullite fibers had good compatibility with the geopolymer matrix. Because the stiffness of fiber was higher than that of the matrix, it was expected that some load would transfer from the geopolymer matrix to the fibers under loading, and hence improve the strength of the matrix.

The fracture mechanism of the sample can also be inferred from the fractal surfaces of the samples. It was found that sections of fibers were detached from the matrix upon fracture (c.f. Figure 4b), which is referred to fiber pullout. It is well known that the additional energy consumed in the process of fiber pullout improves the toughness of the material. These observations indicated that the bonding between the fiber surface and the matrix was desirable for both fiber pullout and load transfer. The strengthening effect was also confirmed by observations during the bending test. The sample with no fibers split into two parts after the test, exhibiting brittle fracture characteristics, while the sample with fibers remained attached.

As shown in Figure 4c, it can also be seen that the cracks were usually accompanied by crack deflection due to fibers, as indicated by the blue arrows. This showed that the fiber/matrix interface was weaker than the matrix. When the crack propagated, it tended to propagate along the weaker fiber/matrix interface. Crack deflection also led to crack branching, as indicated by red arrows in Figure 4c, which further increased the resistance to crack propagation.

### 3.2. Mechanical Properties

**Figure 5:**
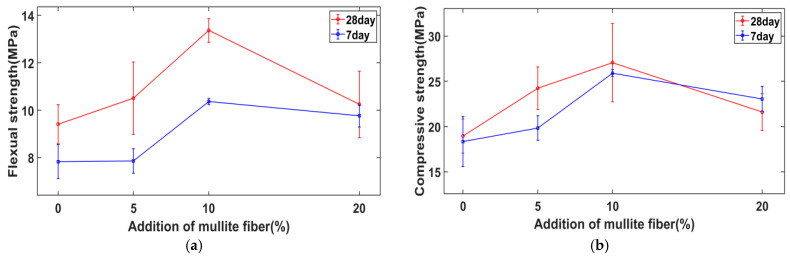
Flexural (**a**) and compressive (**b**) strength of geopolymer composites.

Figure 5 shows the mechanical properties of the samples after 7- or 28-day curing. The maximum compressive strength reached 27 MPa and the maximum flexural strength reached 13 MPa, both of which were achieved with the addition of 10% fibers. Compared with the sample without fiber addition, which had a compressive strength of 18.9 MPa and a flexural strength of 9.4 MPa, they were increased by more than 30%. With the increase in fiber addition, the overall mechanical properties first rose, then decreased, indicating that the addition of 20% fibers had caused some defects in the material. The mechanical properties of the samples cured for 28 days were slightly higher than those of the samples cured for 7 days. The exact data are provided in Table A1 at the end of the paper.

### 3.3. Phase Evolution at Elevated Temperatures

#### 3.3.1. XRD Results

**Figure 6:**
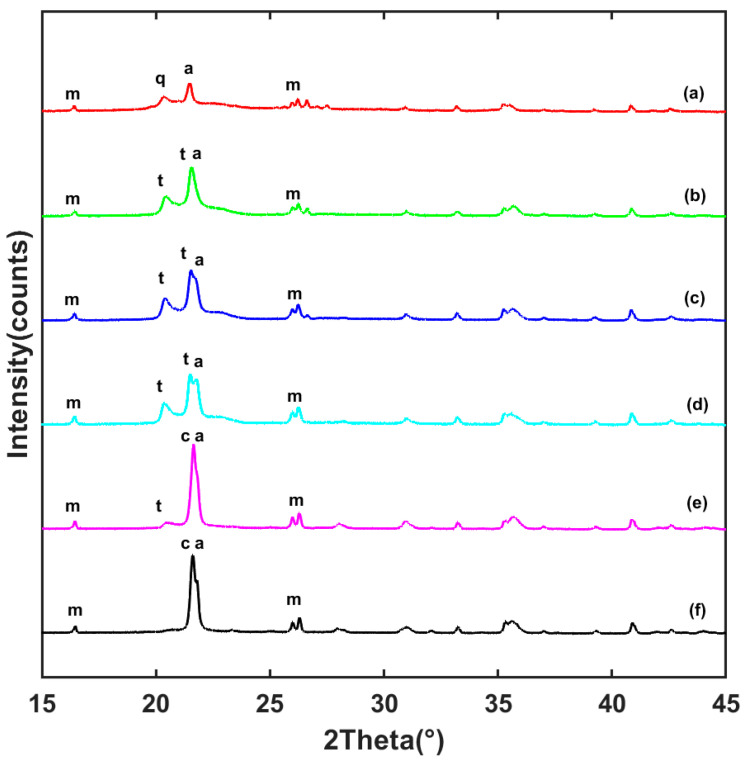
XRD patterns of the composites with 20% fiber addition as prepared or after various heat treatments for 1 h at: (a) room temperature, (b) 1150 °C, (c) 1250 °C, (d) 1350 °C, (e) 1450℃, and (f) 1550 °C. Peak symbols correspond to phases: m—mullite (PDF#15-0776), t—tridymite (PDF#18-1170), c—cristobalite (PDF#39-1425), a—aluminum phosphate (PDF#50-0303), q—quartz (PDF#46-1045).

Figure 6 shows the XRD patterns of geopolymers with 20% fiber after various heat treatments. The sample with 20% fiber was selected to highlight the characteristics of the mullite phase in the sample and verify whether the fiber had an effect on the phase transition of the matrix. Pattern (a) of the sample without any treatment after curing for 7 days revealed that there were quartz-crystalline and amorphous phases. After different heat treatments, the position of the diffraction peak did not change, but the intensity increased. These crystalline phases were identified as quartz and berlinite (aluminum phosphate) phases. After firing at 1150 °C, peaks correlated with tridymite, mullite, and aluminum phosphate phases appearing. After exposure to 1550 °C, the intensity at about 21.5° of the crystal face (101) of cristobalite reached a maximum and tridymite phase disappeared. According to previous studies [25], the geopolymer with complete reaction will show an amorphous phase in XRD patterns. The remaining quartz peaks (~20°) in Figure 6 (line a) indicate that excessive quartz from metakaolinite was not fully consumed by the reaction. It was not found that mullite fiber had any effect on the phase transformation behavior of the matrix after high-temperature treatment.

#### 3.3.2. FTIR Spectra

**Figure 7:**
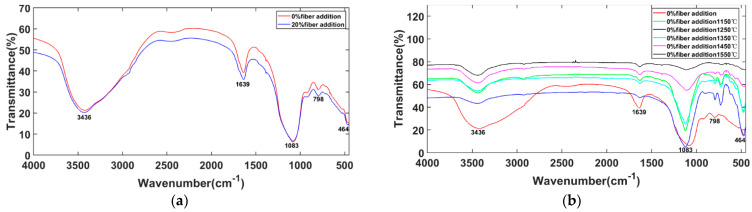
Infrared spectra of geopolymers treated with different fiber ratios and temperatures. (**a**) FTIR spectrums of 0% fibre and 20% fibre without heat treatment; (**b**) FTIR spectrums of 0% fibre with heat treatment at various temperature.

Figure 7a shows the FTIR spectra of the as-prepared geopolymer samples, indicating that the addition of fiber had no significant effect on the curve. The broad absorption band around 3400–3700 cm^−1^ corresponds to the vibration of hydroxyl (OH) groups and water molecules. The band around 1600–1740 cm^−1^ is associated with the adsorbed water and P-O group of stretching and bending vibration. The broad absorption around 950–1300 cm^−1^ corresponds to the stretching vibration of P-O-Si-O-Al-O, which is the main structure of the acid-based geopolymer, as described in previous studies [26,27]. The absorption peaks—798 cm^−1^ for Al-O-Si and 464 cm^−1^ for Si-O bending—showed the presence of mullite and SiO_2_ phases. As shown in Figure 7b, the absorption peaks around 3436 cm^−1^ and 1639 cm^−1^ were reduced, indicating the loss of absorbed water after calcination. After heat treatment at 1450 °C and 1550 °C, there was a significant reduction in the peak (950–1300 cm^−1^) compared to other peaks, indicating the decomposition of P-O-Si-O-Al.

#### 3.3.3. TG-DSC Curves

**Figure 8:**
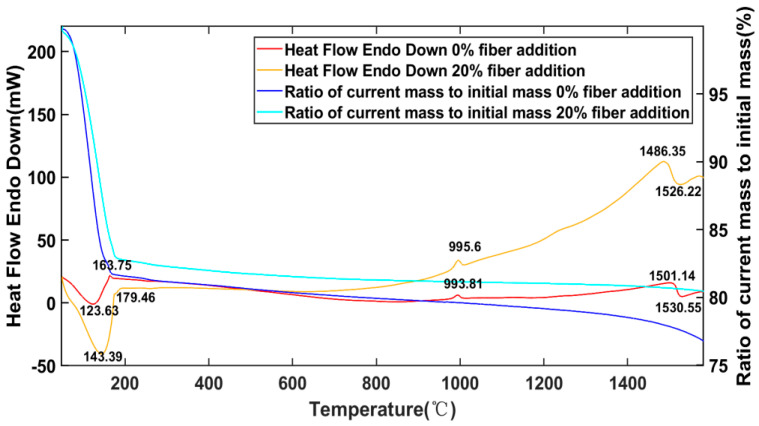
Results of differential thermal analysis of samples.

TG-DSC curves of zero fiber addition and 20% fiber addition samples from 50 °C to 1600 °C were obtained and shown in Figure 8. Since the sample was not dried in advance, the weight loss of 17.27% was detected below 200 °C in 20% fiber addition samples, mainly due to the loss of absorbed water and condensation of hydroxyl groups [28]. There was an obvious endothermic peak in the DSC curve at around 140 °C. A small exothermic peak was observed around 995 °C, related to the phase transformation of quartz to tridymite, as found by the XRD analysis discussed in Section 3.3.1. The exothermic phenomenon detected at 1400 °C to 1500 °C was due to the formation of the mullite phase by consuming SiO_2_ and AlPO_4_. An obvious endothermic peak was detected near 1530 °C. It is believed that this endothermic peak was related to the melting of AlPO₄ phase. After the temperature exceeded 1400 °C, the weight loss of the sample became faster. Again, this was mainly attributed to the decomposition of AlPO_4_ and loss of P_2_O_5_.

#### 3.3.4. SEM Observation

**Figure 9:**
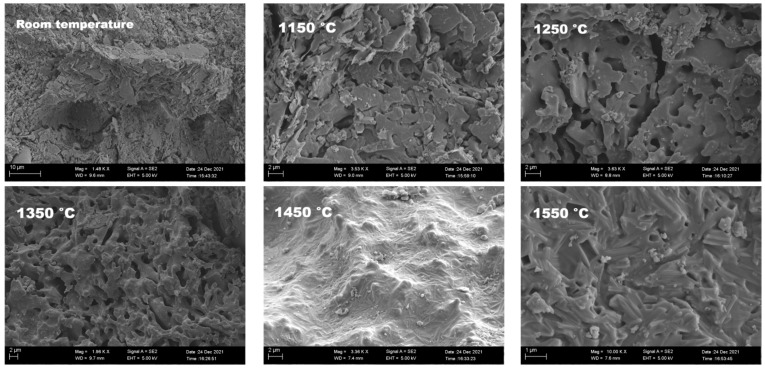
SEM images of samples treated at different temperatures. The heat treatment temperature is indicated on the images.

As shown in Figure 9, the geopolymer at room temperature was mainly composed of a lamellar structure. At 1250 °C, the lamellar structure started to produce some connections. This was due to the formation of a glassy phase because of the presence of alkaline elements in kaolin raw material. When the temperature rose to 1350 °C, the lamellar structure formed a porous structure. After 1450 °C treatment, some fine, needlelike structures were produced on the sample surface, most likely cristobalite according to XRD analysis. When the temperature reached 1550 °C, the needlelike structures evolved into a columnar structure. It is believed that the columnar crystal was mainly due to cristobalite crystal growth in the liquid phase formed by the melting of aluminum phosphate above 1500 °C.

### 3.4. Thermal Properties

**Figure 10:**
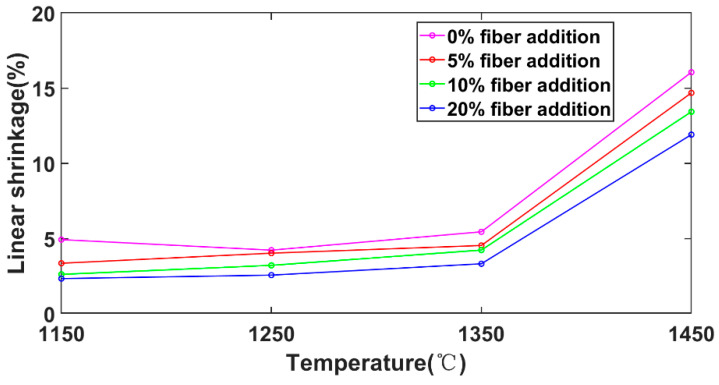
Linear shrinkage at different temperatures.

Through the high-temperature treatment of the sample, the linear shrinkage was obtained, as shown in Figure 10. Each data point is the average of three samples. The linear shrinkage of the sample increased slightly until 1350 °C, reaching about 3%~5%, but suddenly the shrinkage exceeded 10% after treatment at 1450 °C. Generally, the linear shrinkage of the sample with 20% fiber was about 30% lower than that of the sample without fiber addition. The exact data are provided in Table A2 at the end of the paper.

Up to 1350 °C, the sample basically maintain its shape, but some microcracks were observed on the surface. At 1450 °C, the sample underwent serious deformation and cracks appeared. At 1550 °C, the deformation became more serious, even showing signs of flow. The results correlated with the TG-DSC analysis described in Section 3.3.3. The sudden increase in deformation at 1450 °C coincided with the exothermic peak and weight loss between 1400 and 1500 °C, due to the decomposition of the geopolymer chain and AlPO_4_ coinciding with the formation of mullite. The loss of shape at 1550 °C was attributed to the melting of the AlPO_4_ phase, as discussed in previous sections.

**Figure 11:**
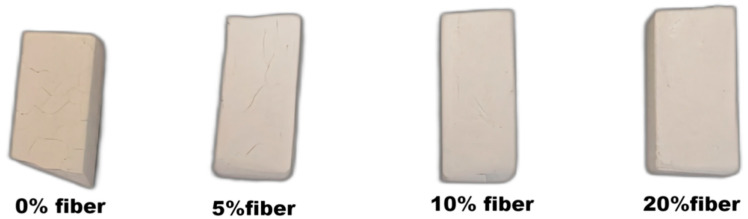
After 1150 °C treatment for 1h, the surface cracks of the sample.

**Figure 12:**
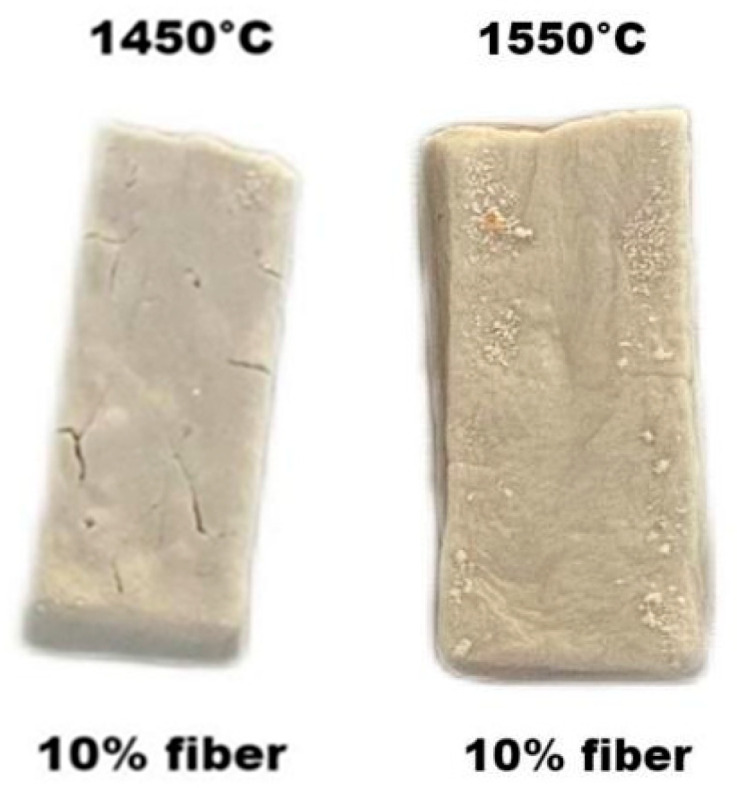
The surface of 10% fiber-added samples after 1450 °C and 1550 °C treatment.

It was noted that not only the linear shrinkage of the sample decreased with the addition of fibers but also the number of cracks on the sample surface decreased and were invisible with the addition of more than 10% fibers, as shown in Figure 11 and Figure 12. The results confirmed that the addition of mullite fibers not only hindered the shrinkage during heat treatment but also improved the fracture resistance of the matrix. This was due to the interlocking of the fibers, which hindered the viscous flow of the material while improving the fracture toughness, described above.

## 4. Conclusions

Mullite fiber has good affinity for phosphate geopolymer and a significant fiber reinforcement effect. The addition of 10% mullite fiber was optimal for compressive/flexural strength, reaching a 28-day compressive strength over 27 MPa and flexural strength over 13 MPa, while the addition of 20% had the strongest inhibitory effect on shrinkage after high-temperature calcination: linear shrinkage of 3.32% after calcination at 1350 °C for 1 h, 30% lower than that without fiber addition.

The main strengthening mechanisms of the fiber were fiber pull-out and fiber deflection effects, indicating desirable fiber/matrix interface bonding.

The polymeric structure of P-O-Si-O-Al-O is the main structure of geopolymer activated by phosphoric acid. When heated above 1350 °C, its main forms are mullite and tridymite/cristobalite of SiO_2_.

Although a certain amount of microcracks appeared in the geopolymer treated at 1350 °C for 1 h, it maintained its shape and the inhibition effect on the crack was more obvious with the increase in fiber content. When the temperature went above 1450 °C, the decomposition and melting of AlPO_4_ became a limiting factor for the composites.

The effect of mullite fibers on a phosphate-based geopolymer has been proved. The obtained temperature resistance means that this composite has the potential to be used as a mold core for casting of low melting point metals, such as aluminum, or as fire-resistant bricks bearing no more than 1350 °C, which is worthy of further study. The author believes that if the content of alkali metals in raw materials is further reduced, the thermal stability of the geopolymer will be improved. Under ideal conditions, the temperature will not exceed the melting point of AlPO_4_.

## Figures and Tables

**Table 1:** Chemical compositions of kaolin and metakaolin.

Raw Materials	Chemical Composition (wt%)								
	${\mathrm{SiO}}_{2}$	${\mathrm{Al}}_{2}O_{3}$	$K_{2}O$	${\mathrm{Fe}}_{2}O_{3}$	${\mathrm{TiO}}_{2}$	PbO	$P_{2}O_{5}$	BaO	MgO
Kaolin	54.37	39.78	2.11	0.916	0.662	0.602	0.445	0.200	0.197
Metakaolin	52.97	41.09	2.08	0.897	0.646	0.591	0.504	0.265	0.218

**Table 2:** Proportion of raw materials used in sample preparation.

Group	Metakaolinite (g)	Phosphoric Acid (85%) (g)	Water (g)	Mullite Fiber (g)
1	100	54	46	0
2	100	54	46	5
3	100	54	46	10
4	100	54	46	20

## Data Availability

Not applicable.

## Appendix A

**Table A1:** Mechanical properties of geopolymer samples.

Addition of Mullite Fiber (wt%)	0	5	10	20
7-day flexural strength (MPa)	7.83 ± 0.72	7.86 ± 0.52	10.37 ± 0.12	9.76 ± 0.47
7-day compressive strength (MPa)	18.33 ± 2.76	19.83 ± 1.37	25.90 ± 0.37	23.03 ± 1.40
28-day flexural strength (MPa)	9.41 ± 0.83	10.51 ± 1.53	13.37 ± 0.50	10.25 ± 1.40
28-day compressive strength (MPa)	18.95 ± 1.89	24.23 ± 2.34	27.07 ± 4.32	21.60 ± 2.05

**Table A2:** Linear shrinkage of geopolymer samples.

	Addition of Fiber (wt%)	0	5	10	20
Temperature (°C)					
1150 °C		4.92%	3.35%	2.61%	2.33%
1250 °C		4.22%	4.02%	3.21%	2.56%
1350 °C		5.44%	4.53%	4.23%	3.32%
1450 °C		16.05%	14.68%	13.42%	11.90%
